# Two Novel Ceramide-Like Molecules and miR-5100 Levels as Biomarkers Improve Prediction of Prostate Cancer in Gray-Zone PSA

**DOI:** 10.3389/fonc.2021.769158

**Published:** 2021-11-19

**Authors:** Maurizia Mello-Grand, Antonino Bruno, Lidia Sacchetto, Simone Cristoni, Ilaria Gregnanin, Alessandro Dematteis, Andrea Zitella, Paolo Gontero, Caterina Peraldo-Neia, Riccardo Ricotta, Douglas M. Noonan, Adriana Albini, Giovanna Chiorino

**Affiliations:** ^1^ Cancer Genomics Laboratory, Fondazione Edo ed Elvo Tempia, Biella, Italy; ^2^ Laboratory of Innate Immunity, Unit of Molecular Pathology, Biochemistry, and Immunology, Istituto di Ricovero e Cura a Carattere Scientifico (IRCCS) MultiMedica, Milan, Italy; ^3^ Department of Mathematical Sciences, Politecnico di Torino, Torino, Italy; ^4^ I.S.B.—Ion Source & Biotechnologies srl, Biotechnology, Bresso, Italy; ^5^ Department of Urology, San Giovanni Battista Hospital of Torino, Corso Torino, Italy; ^6^ Istituto di Ricovero e Cura a Carattere Scientifico (IRCCS) MultiMedica, Milan, Italy; ^7^ Immunology and General Pathology Laboratory, Department of Biotechnology and Life Sciences, University of Insubria, Varese, Italy; ^8^ Unit of Molecular Pathology, Biochemistry, and Immunology, Istituto di Ricovero e Cura a Carattere Scientifico (IRCCS) MultiMedica, Milan, Italy; ^9^ Laboratory of Vascular Cell Biology and Angiogenesis Istituto di Ricovero e Cura a Carattere Scientifico (IRCCS) MultiMedica, Milan, Italy

**Keywords:** SANIST-CIMS, ceramide, miR-5100, prostate cancer, benign prostatic hyperplasia, liquid biopsy, circulating biomarkers

## Abstract

Reliable liquid biopsy-based tools able to accurately discriminate prostate cancer (PCa) from benign prostatic hyperplasia (BPH), when PSA is within the “gray zone” (PSA 4–10), are still urgent. We analyzed plasma samples from a cohort of 102 consecutively recruited patients with PSA levels between 4 and 16 ng/ml, using the SANIST-Cloud Ion Mobility Metabolomic Mass Spectrometry platform, combined with the analysis of a panel of circulating microRNAs (miR). By coupling CIMS ion mobility technology with SANIST, we were able to reveal three new structures among the most differentially expressed metabolites in PCa vs. BPH. In particular, two were classified as polyunsaturated ceramide ester-like and one as polysaturated glycerol ester-like. Penalized logistic regression was applied to build a model to predict PCa, using six circulating miR, seven circulating metabolites, and demographic/clinical variables, as covariates. Four circulating metabolites, miR-5100, and age were selected by the model, and the corresponding prediction score gave an AUC of 0.76 (C.I. = 0.66–0.85). At a specified cut-off, no high-risk tumor was misclassified, and 22 out of 53 BPH were correctly identified, reducing by 40% the false positives of PSA. We developed and applied a novel, minimally invasive, liquid biopsy-based powerful tool to characterize novel metabolites and identified new potential non-invasive biomarkers to better predict PCa, when PSA is uninformative as a tool for precision medicine in genitourinary cancers.

**Graphical Abstract:**
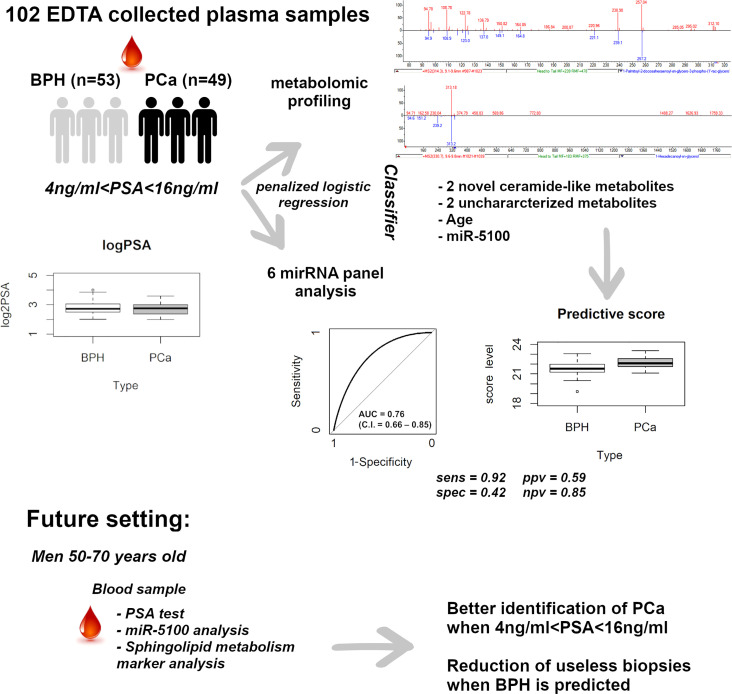
Workflow for sample processing, data analysis, and results from the study.

## 1 Introduction

Prostate cancer (PCa) is among the first two most frequent cancer diagnoses in men (together with lung) and the fifth leading cause of cancer death worldwide (1). PCa incidence and mortality rates are strongly related to age with the highest incidence being seen in men over 65. Currently, the most used test for early diagnosis of PCa is based on prostate specific antigen (PSA), which is prostate, but not cancer specific (2). Digital rectal examination, at the same time, is not a sensitive enough screening test for early PCa. Dutasteride a dual 5α-reductase inhibitor (5-ARI) blocks the conversion of testosterone into its active form dihydrotestosterone (DHT) and reduces prostate volume and prostate-specific antigen (PSA) levels, while increasing urinary flow rate (3). However, this treatment is not always suitable. There is an urgent need for the identification of more accurate diagnostic and prognostic biomarkers, employing minimally invasive procedures, based on liquid biopsy (4).

Application of high resolution and accurate mass spectrometry (HR-MS), due to its analytical performances, experienced high and rapid diffusion in recent years (5). The technology allows to analyze thousands of compounds within a single run, providing information on their identities, especially when combined to tandem mass spectrometry (MS/MS approach). We developed a novel platform that incorporates surface-activated and electrospray (SACI/ESI) ionization source, configured in CIMS (6) operative modality and the SANIST platform, to selectively focalize the metabolomics fraction into the mass spectrometric device. The SANIST platform is particularly useful for studies in biomarker discovery, since it works on different types of biofluids (saliva, urine, serum, and plasma) (6, 7). We successfully used our SANIST platform for biomarker discovery in PCa (6, 7).

miRs are short (19–24 nts), single-stranded non-coding RNAs that regulate gene expression at the post-transcriptional level, either by promoting the cleavage of target mRNAs or by repressing their translation (8). They regulate signaling molecules known to influence the behavior of recipient cells (9). miRs can be easily detected in biological fluids and serve as circulating biomarkers (10). Profiling of circulating miRs has been extensively carried out, with the aim of identifying non-invasive biomarkers to predict the presence of specific tumors, even before they become detectable by diagnostic instrumentations (10). In PCa, there is a deregulation of several miRs that may function as tumor suppressors or oncogenes (11). We recently proposed to combine PSA with two circulating miRs, to better predict PCa, especially when PSA level is between 0 and 4 ng/ml (12).

Here, we focus on the so termed “gray zone”, where it is hard to discriminate PCa from BPH (13). The most accepted definition of gray zone is from 4 to 10 PSA levels. Here, we evaluated men with PSA from 4 to 16. By coupling RT-qPCR analysis of a panel of circulating miRs with the SANIST-CIMS method, we highlight miR-5100, two polyunsaturated ceramide-like ester, and one polysaturated glycerol ester-like structures as potential circulating molecules useful for PCa detection. The role of miR-5100 in tumorigenesis has not been completely elucidated yet; however, in oral squamous cell carcinoma, it mediates cell proliferation and migration (14) and it promotes tumor growth in the lung (15). Ceramides are the central molecules of sphingolipid metabolism, and mediate different kinds of antiproliferative responses (16). An interesting association between sphingolipid metabolism and clinical outcomes has already been proved for some tumors, and this points out to the clinical relevance of sphingolipid-related biomarkers in cancer diagnosis and prognosis (17).

We also propose to combine the analysis of circulating metabolites and miR-5100 with age, to drastically reduce the number of unnecessary invasive biopsies, when PSA is unable to inform on the presence of PCa.

## 2 Materials and Methods

### 2.1 Patient Selection

Plasma samples of 102 males (PSA between 4 and 16 ng/ml) without any previous cancer diagnosis were consecutively collected prior to standard 12-core transrectal ultrasonography-guided biopsy, at the S. Giovanni Battista Hospital of Turin. Forty-nine patients resulted positive for PCa and 53 had all 12 negative biopsies. PCa was labeled GS6, GS7, or GS>7, according to Gleason Score (GS) values; low risk, if GS = 6, PSA < 10, cT < 2b (tumor size, according to clinical TNM staging); intermediate risk, if 10 ≤ PSA ≤ 16 or GS = 7 or cT2b-cT2c; high risk, if GS = 8–9–10 and/or cT3–cT4. Clinically significant tumors comprised intermediate/high-risk PCa. Patients’ characteristics are summarized in **Table 1**. Fourteen out of 102 patients underwent multi-parametric magnetic resonance imaging (mp-MRI). PI-RADs 4 and 5 scores were assigned to suspected images (40%–60% and 60%–90% risk of malignant lesion, respectively). The study was approved by the local Ethics Committee, protocol reference: NC-SERPROS, CE 149/11. All men provided written informed consent with guarantees of confidentiality. Plasma collection, processing, and storage adhered to good practice operations.

**Table 1:** Patient characteristics.

Diagnostic class	BPH (*n* = 53)	PCa (*n* = 49)
Age range	46–83	52–84
Median	66	69
PSA range	4.03–16	4.00–12
Median	6.6	6.72
Low risk		8
Intermediate risk		31
High risk		10
T1c		37
T2		18

Features of patients enrolled in the study, including age range, age median, risk level, and tumor staging, are shown.

### 2.2 Plasma Isolation and Storage

Plasma was isolated from EDTA tube blood samples, within 1 h from collection, with a standard procedure to prevent hemolysis, and stored in 4.5-ml cryovials at −80°C, as previously described (12).

### 2.3 Metabolite Profiling

Metabolomic profiling was performed using the SANIST platform as in (6, 7, 18) with CIMS. For details on chemicals, chromatography, mass spectrometry, and SANIST data elaboration, see **Supplementary Methods** section.

### 2.4 Circulating RNA Extraction

Before extraction, one 220-µl aliquot per sample was centrifuged for 5 min at 1,000*g* at 4°C. Total RNA was isolated with miRNeasy serum/plasma kit (Qiagen) using Exiqon protocol, with MS2-RNA bacteriophage carrier (Roche Diagnostics) to promote RNA precipitation and purification on membranes. cel-miR-39-3p miR mimic spike-in (Qiagen) was added. RNA samples were eluted in 30 μl of nuclease-free water and stored at –80°C.

### 2.5 RT-qPCR

Exiqon miRCURY LNA™ Universal RT microRNA PCR protocol (Exiqon) was followed, starting from 4 μl of total RNA, using cel-miR-39-3p as exogenous normalizer and UniSp6 as internal control for reverse transcription (RT). BioRad CFX96 real-time instrument was used to test six microRNAs on each sample in the same 96-well plate, with three replicated measurements for each test, RT, and real-time negative controls. The six miR tested were as follows: hsa-let-7a, hsa-miR-103a-3p, hsa-miR-21-5p, hsa-miR-320c, hsa-miR-5100, and hsa-miR-874-3p (12). To normalize threshold cycles (Cts), the average Ct (Ct_a) of the three replicates was calculated for each miR and the Ct_a of the miRs in each sample was scaled to have the same cel-miR-39-3p Ct_a, set to the mean cel-miR39-3p Ct_a of all samples. Final normalized values were called Ct_an.

### 2.6 Statistical Analysis

#### 2.6.1 Class Comparison

The *univariate* function of the muma R package was used (19) to identify metabolites differentially expressed in PCa vs. BPH (after scale normalization and batch effect removal pre-processes). For each variable, *univariate* evaluates normality of the distribution in the two groups by means of the Shapiro–Wilk test. For the variables with a normal distribution, Welch’s *t*-test was applied assuming equal variance in the two groups. For the others, the non-parametric Wilcoxon Mann–Whitney test was used.

The analysis was repeated using the limma R package that combines linear models with empirical Bayesian analyses (12).

#### 2.6.2 Classifier

Using log_age, log_PSA, the six Ct_an, and the scaled intensities of seven candidate metabolites (resulting from both muma and limma analyses) as input, a logistic regression model with LASSO penalty was fitted to build a classifier able to discriminate PCa from non-PCa, using the glmnet R package. Fivefold cross-validation was applied to find the best tuning parameter. The estimated log odds ratios of the variables selected by the model were multiplied by their values and then summed to build a score. The ability of the score to correctly classify PCa was measured by the area under the ROC curve (AUC). Sensitivity, specificity, negative predictive value (NPV), and positive predictive value (PPV) were calculated according to a cutoff selected not to misclassify any high-risk PCa. Any patient with score above the cutoff was classified as PCa (positive); any patient with score below the cutoff was classified as BPH (negative). The accuracy of the classifier was then evaluated by summing the true positives and the true negatives and dividing this sum by the total number of patients.

## 3 Results

### 3.1 Workflow and Patient Stratification

**Figure 1:**
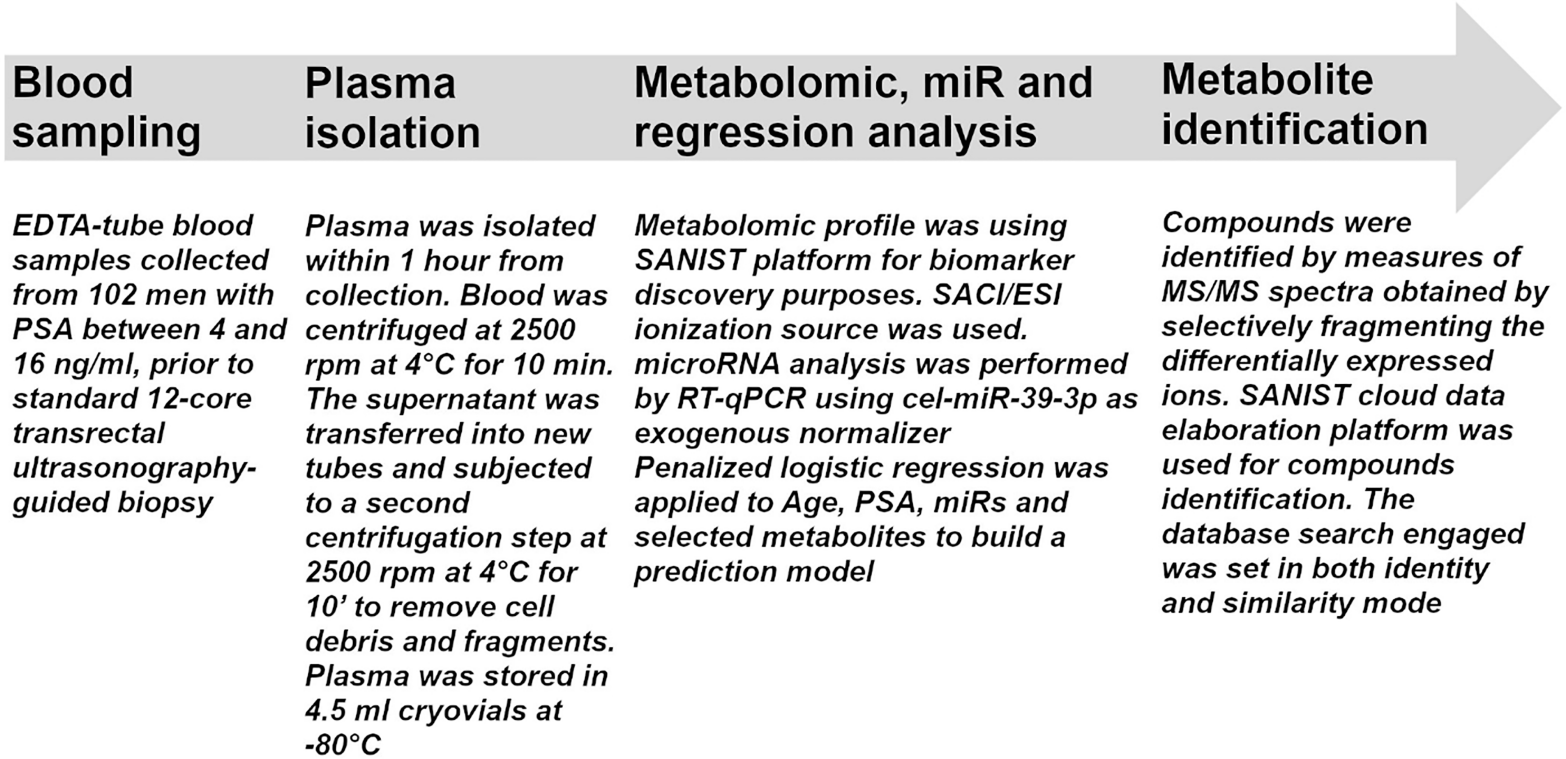
Flow chart for sample processing. The flow chart indicates all the procedures performed in the study from sample collection to metabolite identification.

**Figure 2:**
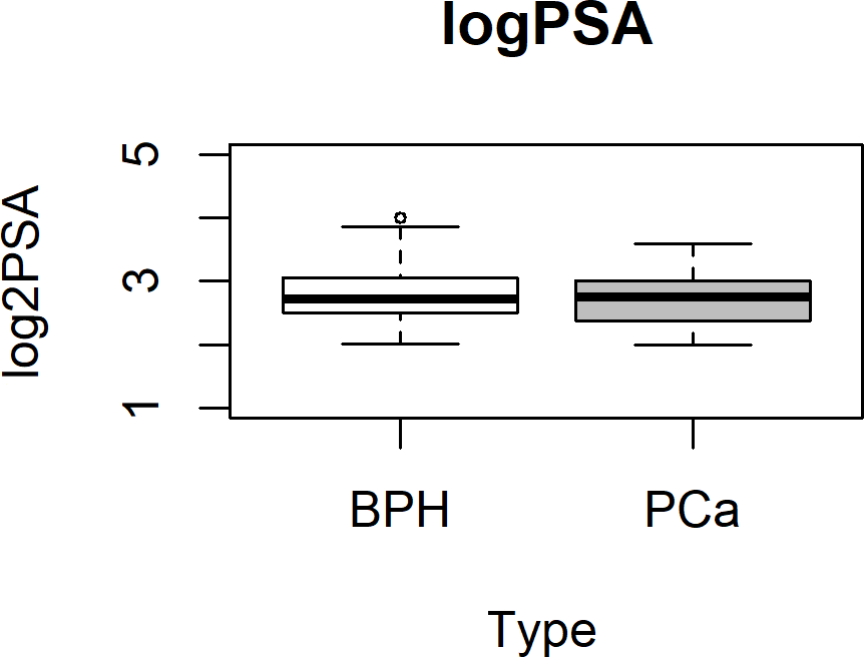
PSA boxplot in BPH and PCa groups. Box plot showing the stratification of samples into the BPH (white box) or PCa (gray box) group. Data are shown as log2 PSA levels (ng/ml). BPH, benign prostatic hyperplasia; PCa, prostate cancer.

Plasma samples from 102 subjects with PSA between 4 and 16 ng/ml, prior to standard 12-core transrectal ultrasonography-guided biopsy, were analyzed. The detailed flow chart of the study is shown in **Figure 1**. Patients’ characteristics are summarized in **Table 1**. **Figure 2** shows the PSA level boxplots for the 53 men with PCa negative biopsy (BPH, benign prostatic hyperplasia) and the 49 patients with PCa positive biopsy (PCa). The figure clearly highlights that PSA levels are totally comparable. This is the reason why new and more accurate biomarkers are needed, to correctly predict the presence of tumor and thus avoid useless and invasive biopsies. A small subgroup of 14 patients underwent mp-MRI: for 5 of them, no suspected lesions were revealed, 7 were classified as PI-RADs 4, and 2 were classified as PI-RADs 5. Four out of eight true BPH did not show suspected lesions and the remaining four were classified as PI-RADs 4. Out of six true PCa, three were PI-RADs 4, two were PI-RADs 5, while one was not identified by mp-MRI.

### 3.2 Selection and Characterization of Candidate Metabolites

**Figure 3:**
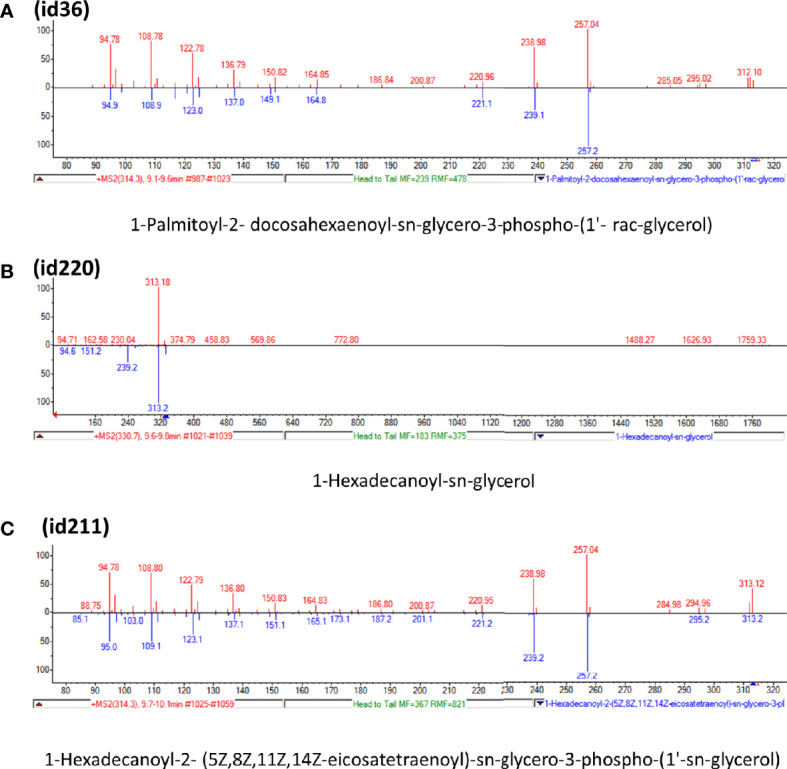
Tandem mass spectra (MS/MS) library similarity match obtained using the NIST library. The tandem mass spectra (MS) of molecules ID36, ID220, and ID211 are shown.

Candidate metabolites were selected by applying two algorithms for univariate analysis. In **Table 2**, compounds with *p*-value less than 0.1 are reported (muma analysis: first two columns from the left; limma analysis: last two columns). Three compounds among the top4 identified by both approaches (ID36, ID211, and ID220) were further studied, to highlight their structural characteristics (**Figure 3**). For details on the process, see **Supplementary Methods** section. The three molecules, similar in structure, are shown in **Table 3**. ID36 and ID211 were classified as polyunsaturated ceramide while ID220 was classified as polysaturated glycerol ester.

**Table 2:** Candidate metabolites differentially expressed between PCa and BPH.

Compound_Muma	Muma *p*-value	Compound	Limma *p*-value
ID249_329_770	0.02	ID36_417_1257	0.03
ID220_441_1036	0.04	ID211_485_1041	0.04
ID211_485_1041	0.04	ID220_441_1036	0.04
ID36_417_1257	0.05	ID249_329_770	0.04
ID114_388_1204	0.06	ID134_471_1438	0.07
ID239_354_1023	0.06	ID109_372_1392	0.07
ID225_326_1520	0.07	ID72_433_1169	0.08
ID55_247_1794	0.07	ID55_247_1794	0.09
ID227_298_1408	0.07	ID202_397_1030	0.09
ID209_329_1145	0.08	ID167_314_1220	0.09
ID224_330_1056	0.09	ID228_343_1201	0.09
ID158_465_1391	0.09		
ID202_397_1030	0.09		
ID167_314_1220	0.09		

The first two columns from the left refer to Muma analysis, while the second two columns refer to limma analysis. Seven compounds are found by both approaches (highlighted in bold).

**Table 3:** Identification of three similar structures within the database.

ID	m/z [M+H]+	Molecular ion identified	Similar structural formula	Relevant scientific notes
36	794	Detected compound with similarities to 1-Palmitoyl-2-docosahexaenoyl-sn-glycero-3-phospho-(1’- rac-glycerol)	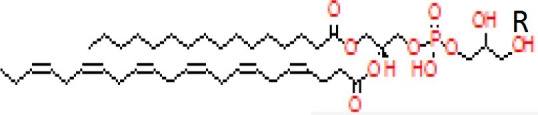	Polyunsaturated ceramide
220	330	1-Hexadecanoyl-sn-glycerol	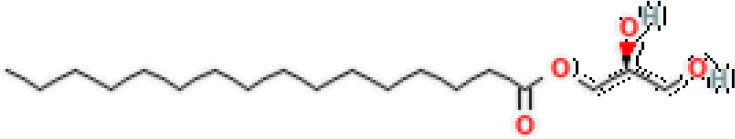	Polysaturated glycerol ester
211	770	Detected compound with similarities to 1-Hexadecanoyl-2-(5Z,8Z,11Z,14Z-eicosatetraenoyl)-sn-glycero-3-phospho-(1’-sn-glycerol)	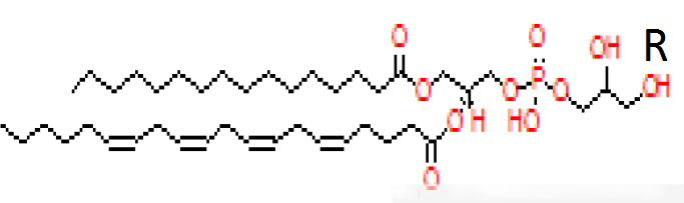	Polyunsaturated ceramide

**Table 3** shows the three molecules, similar in structure, identified in the study.

### 3.3 Prediction Model

**Figure 4:**
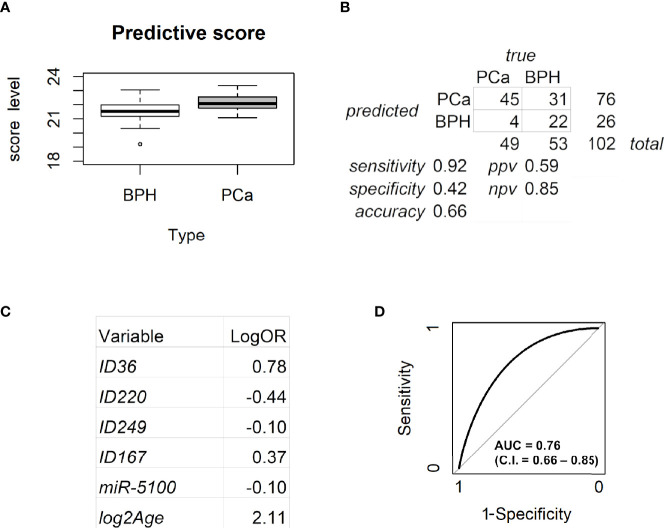
Analysis of the predictive model. **(A)** Box plot for the predictive score discriminating BPH from PCa. Data are shown as the sum of the variable levels multiplied by the regression coefficients as reported in panel C. **(B)** Contingency table corresponding to the cutoff of 21.34 for the predictive score. **(C)** Coefficients of the logistic regression for the selected variables. **(D)** ROC curve of the score obtained by combining age, miR-5100, and four metabolites after penalized logistic regression. BPH, benign prostatic hyperplasia; PCa, prostate cancer.

The seven metabolites resulting from both muma and limma analyses (**Table 2**, in bold) were then included, together with age, PSA levels, and the normalized threshold cycles of the six circulating miRs, as variables in a model to predict PCa (**Figure 4**). Penalized logistic regression was applied to select the variables mostly associated with PCa. Five-fold cross-validation was used to select the best tuning parameter (lambda = 0.0336), which minimized the mean cross-validated error (1.3578), and to fit the model. Combining age, miR-5100, and four metabolites, a score was built (**Figure 4A**). Selecting 21.34 as cutoff, the accuracy of the predictive score was 0.66 (with sensitivity of 0.92, specificity of 0.42, ppv of 0.59, and npv of 0.85, as shown in **Figure 4B**). No high-risk tumor was misclassified and 22 over 53 BPH were correctly classified, reducing by 40% the false positives of PSA in this gray-zone range. **Figure 4C** shows the estimated log odds ratio (logOR) of the selected variables. Age, ID36, ID167, and miR-5100 are positively associated with PCa risk (the minus sign of miR-5100 logOR means that decreasing Ct—and thus increasing expression—increases PCa risk), while ID220 and ID249 are negatively associated. PSA was not selected by the model. The metabolites with higher weight are ID36 and ID220, representing a novel polyunsaturated ceramide and a novel polysaturated glycerol ester, respectively (**Table 3**). The first is more expressed in PCa and the second in BPH. The ROC curve AUC of the predictive score is 0.76 (C.I. = 0.66-0.85; **Figure 4D**).

Considering the 14 patients who underwent mp-MRI, four out of eight true BPH were correctly classified by the model while the remaining four were not, whereas all six true PCa were correctly identified. Therefore, within this very small subgroup of samples, our model reached 100% sensitivity, outperforming mp-MRI, and 50% specificity, as for mp-MRI.

## 4 Discussion

The identification, standardization, and validation of more precise tools to trace relevant and/or discover novel biomarkers in PCa, employing minimally invasive procedures, still represent a major urgency and a clinical unmet need.

Untargeted metabolomics represents a useful approach to analyze body fluids, to detect novel circulating biomarkers. With the aim of identifying metabolites potentially useful to discriminate PCa from BPH, we analyzed plasma of subjects with PSA ranging from 4 to 16 ng/ml, within a cohort of consecutively recruited patients who underwent prostate biopsy. The two diagnostic groups (49 PCa and 53 BPH) were homogeneous in terms of size, age, and PSA values. Class comparison yielded a list of candidate metabolites potentially able to discriminate between the two groups. The same samples were also subjected to RT-qPCR analysis of a panel of six circulating miRs previously derived from miR profiling of a discovery cohort analyzed by our group (12).

By coupling CIMS ion mobility technology with SANIST, we were able to reveal three new structures, two similar to polyunsaturated ceramide and one to polysaturated glycerol ester, among the most differentially expressed compounds in PCa vs. BPH.

Ceramide, the central molecule of sphingolipid metabolism, can mediate various antiproliferative responses. Circulating levels of bioactive sphingolipids may represent novel non-invasive cancer biomarkers and have already been correlated with patient survival and treatment response in different tumor types (17).

In PCa, ceramide induces apoptosis (20), and various molecules may upregulate ceramide in prostate tumor cells (21, 22). Ceramide is a component of a three-lipid signature (ceramide, sphingomyelin, and phosphatidylcholine) associated with poor prognosis in castration-resistant PCa (23). An association between biochemical recurrence (PSA increase after radical prostatectomy) and ceramide, along with acyl-carnitines (24), which we and others previously reported as biomarkers (6, 25), has also been observed. There are many ceramide analogues with known biological activities (26–28), but no information is available on polyunsaturated ceramides.

To build a model able to predict PCa, we applied penalized logistic regression using both metabolomics and microRNA variables, together with age and PSA. As expected, PSA was not selected by the model as an informative variable. Only hsa-miR-5100 was retained, together with four metabolites and age, the selected variable with highest weight (**Figure 4C**). The prediction model had an AUC of 0.76, considerably high in the PSA range considered. Other proposed PSA-related biomarkers, the four-kallikrein panel (4K) and the Prostate Health Index (PHI), evaluated on 531 men with PSA 3–15 ng/ml undergoing first-time prostate biopsy, thus on a quite similar context, showed AUCs of 0.69 and 0.70, respectively, when predicting any-grade PCa (29).

Among the polyunsaturated characterized compounds, ID36 and ID220 were the most informative, whereas ID211 was not selected. Interestingly, its profile is very similar to ID220 (Pearson correlation coefficient = 0.8) whereas its structure is very similar to ID36 (**Figure 3**). Two other uncharacterized compounds (ID249 and ID167), that deserve future identification, were included in the predictive model, although with lower logOR (**Figure 4C**).

To date, little is known about miR-5100; nevertheless, it is gaining considerable interest, especially in cancer: its upregulation has been associated with tumor growth (30), invasion, and metastasis promotion (14, 31) and several publications proposed it as a candidate biomarker to predict prognosis (recurrence and/or survival) in classifiers, along with other miRs and/or clinical parameters (31–33). Examples of downregulation in cancer tissue (e.g., pancreatic cancer) are reported as well (34). There are no studies related to PCa. In liquid biopsy, few studies are available: Shi et al. observed the upregulation of serum miR-5100 in oral squamous cell carcinoma patients (31), whereas Yuan and colleagues (35) found an association between low expression of miR-5100 in plasma and the risk of childhood acute lymphocytic leukemia. miR-5100 was also proposed as a biomarker for two autoimmune diseases (lupus erythematosus and Sjögren’s syndrome) (36, 37). Very recently, Hua-Ping Liu and collaborators (38) proposed a highly performant pairwise model composed of five circulating miRNAs coupled to miR-5100 and miR-1290, and found miR-5100 upregulation in three independent big serum PCa cohorts. Its mechanisms of action and targets may be tissue and disease stage dependent: in pancreatic cancer, it was proposed as anti-metastatic miR, through PODXL silencing (34). On the contrary, hypoxic BMSC-derived exosomal miRs (including miR-5100) promote metastasis of lung cancer cells *via* STAT3-induced EMT (31). In lung cancer, miR-5100 overexpression supports tumor growth (30) and increases Cisplatinum resistance of lung cancer stem cells (15) by inhibiting Rab6, a protein located at the Golgi body that regulates membrane traffic from the Golgi apparatus towards both the endoplasmic reticulum and the plasma membrane. Interestingly, the Golgi apparatus is the site at which ceramide can be further modified to sphingolipids, and this suggests a possible link between miR-5100 and the polyunsaturated ceramide-like similar metabolites we identified.

The present study is limited by the small number of samples, but is focused on a specific PSA range where there is about 50% chance of getting a PCa diagnosis. Patients have been consecutively enrolled in one center, ensuring homogeneous procedures of sample collection and processing, but requiring an external validation step. Aware of these limits, we have already started a prospective study involving three centers and 700 patients aged 50–69 years, in order to validate our findings.

In conclusion, the combination of novel circulating biomarkers proposed in this study may help to reduce the number of invasive biopsies when PSA is uninformative on the presence of PCa.

## Data Availability Statement

The original contributions presented in the study are included in the article/**Supplementary Material**. Further inquiries can be directed to the corresponding authors.

## Ethics Statement

The study was approved by the local Ethics Committee, protocol reference: NC-SERPROS, CE 149/11. The patients/participants provided their written informed consent to participate in this study.

## Author Contributions

Project coordination/supervision: AA, DN, and GC. Conception and design: AA, DN, GC, and SC. Acquisition of data: SC, GC, MM-G, LS, CP-N, AD, AZ, and PG. Analysis and interpretation of data: MM-G, AB, LS, SC, CP-N, DN, AA, GC, and PG. Clinical discussion: RR. Manuscript drafting: AB, SC, DN, AA, and GC. Statistical analysis: MM-G, LS, SC, IG, and GM. All authors contributed to the article and approved the submitted version.

## Funding

The study was supported by Compagnia di San Paolo (Grant no. 5086-2013.1535 to GC), Reale Foundation (Network for People Grant to GC). AB is funded by an AIRC MFAG 2019-ID 22818 grant and by a Cariplo Foundation id-2019-1609 grant. DN is funded by Progetti di Rilevanza Internazionale (PRIN) 2017, grant 2017NTK4HY. AB, AA, and DN are supported by the Italian Ministry of Health, Ricerca Corrente - IRCCS MultiMedica.

## Conflict of Interest

Author SC was employed by Ion Source & Biotechnologies srl.

The remaining authors declare that the research was conducted in the absence of any commercial or financial relationships that could be construed as a potential conflict of interest.

## Publisher’s Note

All claims expressed in this article are solely those of the authors and do not necessarily represent those of their affiliated organizations, or those of the publisher, the editors and the reviewers. Any product that may be evaluated in this article, or claim that may be made by its manufacturer, is not guaranteed or endorsed by the publisher.
