# Supplementary material for: Two Novel Ceramide-Like Molecules and miR-5100 Levels as Biomarkers Improve Prediction of Prostate Cancer in Gray-Zone PSA
Source: Front Oncol. 2021 Nov 19;11:769158. doi: 10.3389/fonc.2021.769158 (PMC8640468; doi:10.3389/fonc.2021.769158)
Supplement: Supplementary file 1 [file DataSheet_1.docx]

Supplementary methods

*Chemicals*

Acetonitrile (CH_3_CN), bi-distilled water (H_2_O), Dimethylsulfoxide (DMSO) and formic acid (HCOOH) were purchased from Sigma Aldrich (Milan, Italy). The solid fraction was then dissolved using 70 μl of H_2_O/CH_3_CN 80/20 solution containing 0.1% of Formic Acid. Each sample was prepared and analsed in triplicate.

*Chromatography*

To analyse the selected analytes, an Ultimate 3000 UPLC (Thermo Fisher, San Jose, CA, USA) LC apparatus was employed to obtain analyte separation before MS analysis. A Trascend System HPLC (Thermo Fisher, San Jose, CA, USA) was used for separation. The mobile phases were: A) H_2_0 + 0.1% HCOOH and B) CH_3_CN. A binary gradient was used: 2% of B was maintained for 5 minutes, in 10 minutes B was raised to 30%, in other 20 minutes B was brought to 80% and maintained for 5 minutes, then 2% of B was reached in 1 minute and the column was re-equilibrated in starting conditions for 9 minutes. The chromatographic flow was 0.50 mL/minute. The injection volume was 20 µL.

*Mass spectrometry*

Product analysis was performed using LTQ XL ORBITRAP mass Spectrometer (Thermo Fisher, San Jose, USA) coupled to Heated ESI a Heated SACI-ESI source (described in (1)) and operated in CIMS alternate mode (2). Heated ESI capillary voltage was 2750 Volt, dry gas: 2L/min, Nebulizer: 60 psi and Temperature: 40°C. Tandem MS experiments were performed in Collision Induced Dissociation (CID) conditions using He as collision gas and a collision energy of 35% of its maximum value (5 V peak to peak). Heated SACI-ESI was set in alternate CIMS conditions to focalize ion cloud containing low molecular MW and medium/High one. The former conditions were capillary voltage was 100 Volt, SACI surface voltage was 47 V, Dry gas: 0.5L/min, Nebulizer: 70 psi and Temperature: 40°C. The latter conditions where capillary voltage was 1500 Volt, SACI surface voltage was 47 V, Dry gas: 2L/min, Nebulizer: 70 psi and Temperature: 40°C. Tandem MS experiments were performed in Collision Induced Dissociation (CID) conditions using N_2_ as the collision gas.

*SANIST data elaboration platform*

SANIST data elaboration platform has been described in detail previously (3-5). Briefly, the platform is composed by LC coupled to a mass spectrometer provided with a SACI/ESI/ionization source. The obtained data are then transferred in a cloud platform to be processed for obtaining metabolomic expression values.

*Selection of candidate metabolite*

In the preliminary experiments, we adjusted the setting CIMS (5) to increase the metabolite signal intensity m/z (optimized m/z zone: 50 - 800 Th). The method was set to switch between low and high molecular weight each 0.2 sec. The ion mobility nitrogen gas under 3 L/min does not select any charged cloud and every cloud containing different molecular weight and charged analytes is focalized vs the first mass spectrometer ion vacuum region at 0.47 tor. The solvent clouds containing the low m/z ratio analytes are selected using the 150 V focusing voltages. In fact, in these conditions the solvent cloud containing the low singly species are better focalized to the MS vacuum region while the focusing voltage is not optimal to efficiently direct the high m/z in the MS entrance hole.

1. Rubert J, Zachariasova M, Hajslova J. Advances in high-resolution mass spectrometry based on metabolomics studies for food--a review. Food Addit Contam Part A Chem Anal Control Expo Risk Assess **2015**;32(10):1685-708 doi 10.1080/19440049.2015.1084539.

2. Arzoni A, Bernardi LR, Cristoni S. In-source cloud ion mobility mass spectrometry. Rapid Commun Mass Spectrom **2015**;29(7):690-4 doi 10.1002/rcm.7136.

3. Albini A, Briga D, Conti M, Bruno A, Farioli D, Canali S*, et al.* SANIST: a rapid mass spectrometric SACI/ESI data acquisition and elaboration platform for verifying potential candidate biomarkers. Rapid Commun Mass Spectrom **2015**;29(19):1703-10 doi 10.1002/rcm.7270.

4. Albini A, Bruno A, Bassani B, D'Ambrosio G, Pelosi G, Consonni P*, et al.* Serum Steroid Ratio Profiles in Prostate Cancer: A New Diagnostic Tool Toward a Personalized Medicine Approach. Front Endocrinol (Lausanne) **2018**;9:110 doi 10.3389/fendo.2018.00110.

5. Cristoni S, Dusi G, Brambilla P, Albini A, Conti M, Brambilla M*, et al.* SANIST: optimization of a technology for compound identification based on the European Union directive with applications in forensic, pharmaceutical and food analyses. J Mass Spectrom **2017**;52(1):16-21 doi 10.1002/jms.3895.
